# Critical barriers to sustainable capacity strengthening in global health: a systems perspective on development assistance

**DOI:** 10.12688/gatesopenres.13632.2

**Published:** 2023-01-18

**Authors:** Barbara Knittel, Amanda Coile, Annette Zou, Sweta Saxena, Logan Brenzel, Nosa Orobaton, Doris Bartel, Cecilia Abimbola Williams, Rose Kambarami, Dipak Prasad Tiwari, Ishrat Husain, Godfrey Sikipa, Jane Achan, John Ovuoraye Ajiwohwodoma, Banny Banerjee, Dyness Kasungami

**Affiliations:** 1 JSI Research & Training Institute, Inc., Arlington, VA, 22202, USA; 2 Global ChangeLabs, Portola Valley, CA, 94028, USA; 3 United States Agency for International Development, Washington, DC, 20523, USA; 4 Bill & Melinda Gates Foundation, Seattle, WA, 98109, USA; 5 Independent Researcher, Washington, District of Columbia, USA; 6 Independent Consultant, Abuja, Nigeria; 7 University of Zimbabwe, Harare, Zimbabwe; 8 Bagmati Province Health Office, Ministry of Health, Chitwan, Nepal; 9 COMPRE Health Services, Harare, Zimbabwe; 10 Malaria Consortium, Kampala, Uganda; 11 Federal Ministry of Health, Abuja, Nigeria

**Keywords:** Development assistance for health, capacity strengthening, system design, donor funding reform, donor-recipient relationships, aid reform, technical assistance, health systems strengthening, Reimagining TA, critical shifts

## Abstract

**Background: **Development assistance for health (DAH) is an important mechanism for funding and technical support to low-income countries. Despite increased DAH spending, intractable health challenges remain. Recent decades have seen numerous efforts to reform DAH models, yet pernicious challenges persist amidst structural complexities and a growing number of actors. Systems-based approaches are promising for understanding these types of complex adaptive systems. This paper presents a systems-based understanding of DAH, including barriers to achieving sustainable and effective country-driven models for technical assistance and capacity strengthening to achieve better outcomes

**Methods: **We applied an innovative systems-based approach to explore and map how donor structures, processes, and norms pose challenges to improving development assistance models. The system mapping was carried out through an iterative co-creation process including a series of discussions and workshops with diverse stakeholders across 13 countries.

**Results:** Nine systemic challenges emerged: 1) reliance on external implementing partners undermines national capacity; 2) prioritizing global initiatives undercuts local programming; 3) inadequate contextualization hampers program sustainability; 4) decision-maker blind spots inhibit capacity to address inequities; 5) power asymmetries undermine local decision making; 6) donor funding structures pose limitations downstream; 7) program fragmentation impedes long-term country planning; 8) reliance on incomplete data perpetuates inequities; and 9) overemphasis on donor-prioritized data perpetuates fragmentation.

**Conclusions: **These interconnected challenges illustrate interdependencies and feedback loops manifesting throughout the system. A particular driving force across these system barriers is the influence of power asymmetries between actors. The articulation of these challenges can help stakeholders overcome biases about the efficacy of the system and their role in perpetuating the issues. These findings indicate that change is needed not only in how we design and implement global health programs, but in how system actors interact. This requires co-creating solutions that shift the structures, norms, and mindsets governing DAH models.

## Introduction

Low-income countries (LICs) have traditionally relied on funding and technical support from bilateral donors, centrally funded mechanisms, and philanthropic foundations for implementing health programs ^1, 2^. This donor-recipient dichotomy has created power imbalances that favor donors and has led to many well-documented challenges, including a misalignment between donor and recipient country priorities, short-term and often complex funding structures and processes, the introduction of parallel systems, and a “fly-in fly-out” approach to technical assistance (TA) that can undermine capacity strengthening efforts and sustainability ^1, 3^. These factors have contributed to inefficiencies and are believed to have stifled health program co-design and capacity strengthening in ways that hamper local ownership, sustainability, and progress toward desired health outcomes ^4, 5^. The coronavirus disease (COVID-19) pandemic has amplified many of these inefficiencies and exposed gaps in the development assistance system that hinder response efforts and ^6– 8^, in some instances, further fragment health services ^9^.

These challenges are broadly recognized across actors in the global health community, and calls for reforming development assistance are not new. In 2005, donors and recipient countries came together and drafted the Paris Declaration on Aid Effectiveness ^10^. Since then, many other aid effectiveness initiatives have been launched, including the International Health Partnership plus (2007) ^11^, the Accra Agenda for Action (2008) ^12^, the Busan Partnership for Effective Cooperation (2011) ^13^, the Global Partnership for Effective Development Cooperation (2011), the Addis Ababa Action Agenda (2015), and the Universal Health Coverage 2030 Global Compact (2017). The 2030 Agenda for Sustainable Development also underscores the continued desire for a better aid model ^14^.

Despite these global efforts, there has been little progress toward a more sustainable and effective model for delivering development assistance in the health sector ^14^. For instance, in 2017, aid spending on reproductive, maternal, newborn, and child health reached $15.6 billion ^15^. Yet, despite perennial increases in aid spending, the annual death toll for mothers and children remains unacceptably high, and many more suffer from illness and disability ^15, 16^. The global health community is still grappling with the same fundamental challenges, including pernicious fragmentation, donor proliferation, and misalignment of donor-recipient priorities ^6, 17, 18^. While there has been growing rhetoric around country-owned and adaptive development assistance, models and cases of more sustainable approaches are limited and do not account for the context of COVID-19 or similar shocks to the health system ^19– 21^.

The challenges underlying development assistance reform are complex and depend on broader health system context. Health systems can be characterized as complex adaptive systems such that they are composed of a diverse set of individual actors and elements that are self-organized around a shared purpose, and continuously interact in a non-linear and non-deterministic manner ^22, 23^. Over the past 20 years, stakeholders, networks, boundaries, and interests governing health systems and affecting development assistance have expanded ^6, 24^. The literature points to persistent development assistance challenges, including evolving political will and constituent interests; a changing donor landscape that now includes more multilateral, private sector, and south-south partnerships; an over-reliance on technical solutions and constraints imposed by donor systems and requirements; time-bound global goals that put pressure on achieving results over longer-term systems change; broader geopolitical and economic contexts; and prevailing power asymmetries ^25– 27^. These complexities can be understood as a ‘wicked challenge,’ first introduced by Rittel and Webber ^28^, such that even defining and locating the right problems and knowing what distinguishes an observed condition from a desired one poses an intractable challenge. Overcoming such challenges requires a deeper understanding of the linkages, relationships, interactions, and behaviors that characterize an entire system ^29, 30^.

### A systems approach to development assistance reform

There have been calls to action by large international organizations such as the United Nations and World Economic Forum to inspire more transformative approaches to overcome intractable global challenges. In 2009, the World Health Organization and the Alliance for Health Policy and Systems Research issued a guide for using a “systems thinking” approach for health systems strengthening ^30^, arguing that the approach can “open powerful pathways to identifying and resolving health system challenges, and as such is a crucial ingredient for any health system strengthening effort.”

Systems-based approaches (e.g., systems thinking, systems transformation, systems innovations) seek to examine these complexities by analyzing drivers and causal relationships, and identifying key intervention points for system change ^31^. System Acupuncture ^32^, as one such methodology, uses a set of desired outcomes to frame the exploration of complex interconnecting system drivers and, through a facilitated co-creation process with a diverse group of stakeholders, arrives at an understanding of why current outcomes are produced. Having gained a perspective on underlying dynamics, the approach guides stakeholders to identify opportunities for transforming the behavior of the system to achieve scaled and sustained outcomes.

### Purpose of this paper

Using the System Acupuncture method, this paper presents a systems-based understanding of development assistance for health, including barriers to achieving better, more sustainable country-driven models for TA and capacity strengthening to achieve better outcomes. This paper seeks to inform the ongoing discussion on development assistance reform and support the identification of key actions at project, country, regional, and global levels that facilitate investments to sustain local development and build resilient, equitable health systems.

This work took place under the Inter-agency Working Group (IAWG) for Capacity Strengthening initiative. The IAWG is composed of representatives from the Bill & Melinda Gates Foundation, United States Agency for International Development, and The World Bank. The IAWG seeks to harness its collective power to improve financial investments in capacity strengthening to foster more resilient health systems and sustained health outcomes. JSI Research & Training Institute, Inc. and Global ChangeLabs are the IAWG secretariat.

## Methods

A systems approach, guided by the System Acupuncture method ^32^, was employed to create an understanding of capacity strengthening in the context of development assistance. This paper focuses on the system diagnostic component of the System Acupuncture process, and was conducted through an iterative co-creation process over 12-months. Participants in this work represented diverse stakeholder groups, including the IAWG members and additional representatives from their institutions, as well as country-based government, civil society, implementers, private sector, and academia. The secretariat and IAWG members used purposive sampling to identify actors within their networks who could bring diverse perspectives on how health funding and TA is structured at various levels and how donor processes, models, and norms constrain or amplify health system capacity strengthening and sustainable health outcomes. Participants were selected to ensure diversity in background, institutional affiliation, geography and perspectives in order to co-create a systems view.

Given the importance of equity in developing insights, the co-creation process aimed to reduce power imbalances between participants by establishing community norms and anonymizing inputs during working sessions.

### Defining the system

In this work, the “system” is defined as the dynamic network of causal relationships linking the actors, behaviors, prevalent drivers, and situational contexts that influence development assistance for health outcomes.

**Figure 1.**
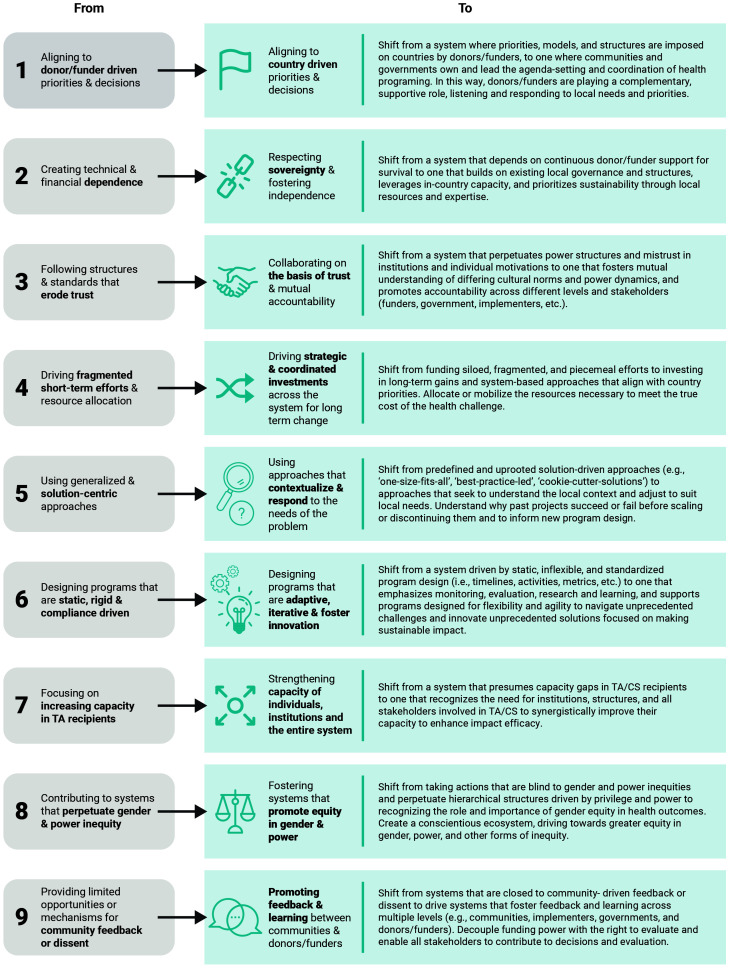
Critical Shifts for Capacity Strengthening.

The Critical Shifts for Capacity Strengthening (Critical Shifts for CS), highlighted in Figure 1, was a guiding framework for this initiative and represents a vision for the desired outcomes of the system in focus. This framework was derived from the Re-Imagining Technical Assistance (RTA) for Maternal, Neonatal, and Child Health and Health Systems Strengthening project and adapted under this initiative to focus on capacity strengthening and highlight important power and gender dimensions missing from the original framework ^19^.

This initiative hypothesizes that by achieving the Critical Shifts for CS, a more sustainable and equitable development assistance model will emerge and strengthen health system capacity to deliver desired health outcomes.

In line with the IAWG’s purpose to improve donor ^i^ investments in capacity strengthening, we narrowed the boundaries of our system to defining how donor structures, processes, behaviors, and norms inhibit the realization of the Critical Shifts for CS.

### Systems diagnostic process

Using the Critical Shifts for CS as our desired outcome of the system, we co-created a visual depiction of the systemic barriers to progress toward this vision. Steps included:

**Mapping the system:** Using the System Acupuncture technique called ‘reverse causal chain mapping,’ we developed an initial system map based on insights drawn from the RTA initiative, conversations with IAWG members and broader representatives from their organizations, background literature, and secretariat expertise. To iterate on the initial map, we convened 41 participants from 13 countries for a two-day virtual co-creation workshop in April 2021. ^ii^ Participants were taken through a process to examine the assumptions in the initial system map and identify additional drivers. Using the collaborative online white-boarding tool Miro, participants worked in small groups to identify and map donor and country interactions that pose barriers to achieving the Critical Shifts for CS.

**Figure 2.**
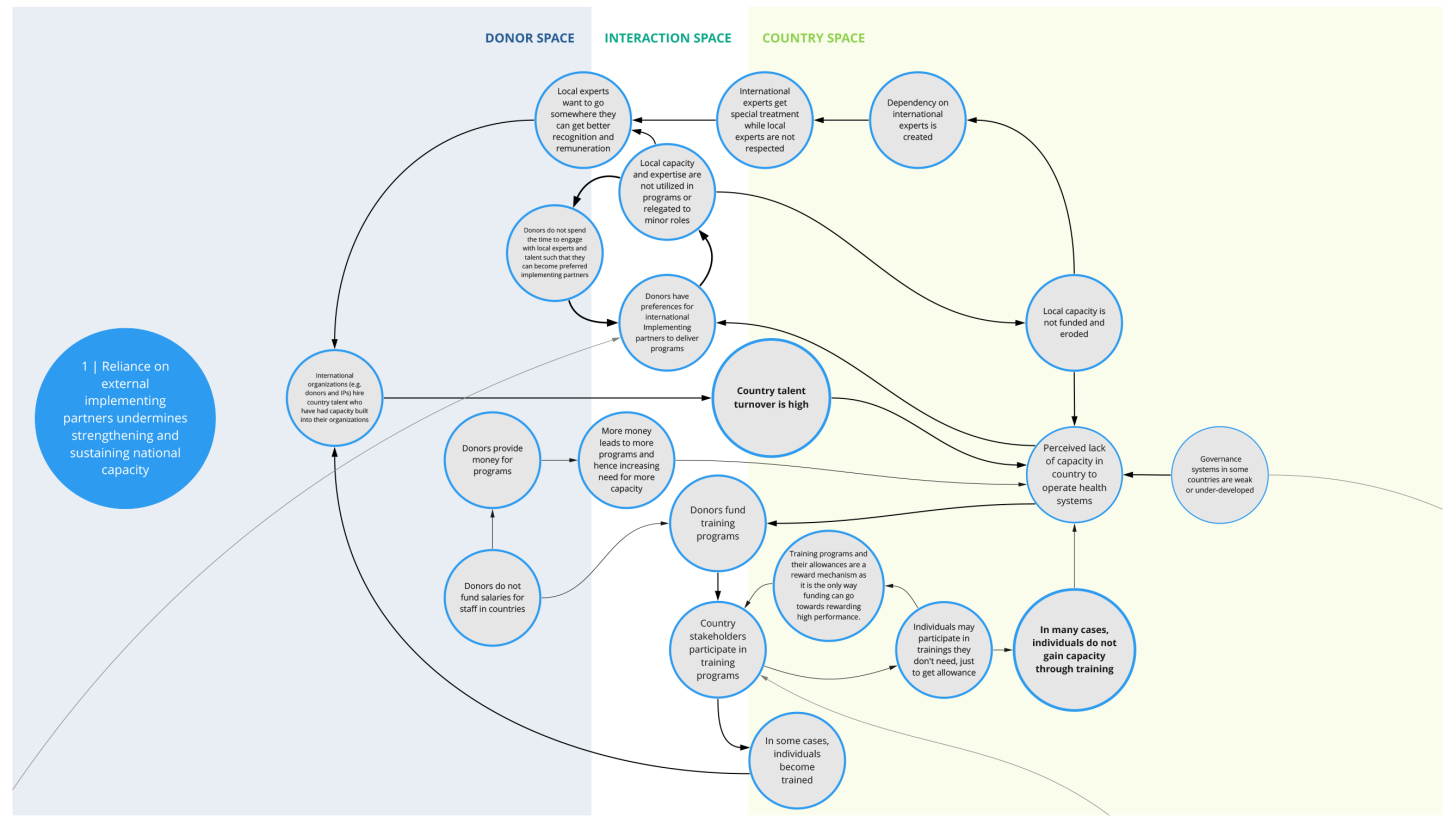
Syndrome 1: Reliance on external implementing partners undermines strengthening and sustaining national capacity.

**Synthesizing outputs and identifying key system ‘syndromes:’** The secretariat and IAWG members (“the team”) synthesized workshop outputs by reviewing and grouping key drivers identified by participants into broader challenge areas. In Systems Acupuncture, sets of drivers that occur concurrently and have an identifiable pattern of characteristics are referred to as ‘syndromes.’ The team derived a set of nine syndromes that represent systemic challenges to achieving the Critical Shifts for CS and arranged workshop insights into a set of interconnected system syndrome maps ( Figure 2– Figure 10).

**Refinement of the system syndromes:** The syndrome maps continued to be refined as insights and clarifications emerged from monthly discussions with IAWG members, dialogue sessions with a broader group of representatives from IAWG organizations, and ad hoc engagement of workshop participants via email. We conducted additional discussions and analyses to explore gender dynamics across the syndromes; this is reported in a separate forthcoming manuscript.

### Ethical approval

This work was carried out through a facilitated co-creation process, including one workshop and a series of discussions that elicited benign and anonymous feedback from selected participants. To ensure privacy and confidentiality, participant insights were gathered anonymously via Miro. Additionally, consent to collect inputs and record sessions was obtained from all participants at the start of the workshop sessions. All inputs were collected and analyzed with complete anonymity and are therefore unable to be linked to a single individual. The project team did not seek ethical approval because we determined the activities were exempt given that they did not constitute human subjects research as described under US HHS regulation 45 CFR 46(e) (1) and aligned with exemption requirements outlined under 45 CFR 46.104 (d)(3a) and (d)(3b).

## Results

### System syndromes

The nine syndromes discussed below represent systemic barriers to achieving the Critical Shifts for CS. The accompanying maps ( Figure 2– Figure 10) illustrate the multi-dimensional causal relationships between individual drivers and display feedback loops (i.e., sets of drivers that reinforce each other) within and between syndromes. The syndromes, in no particular order, are: 1) reliance on external implementing partners undermines strengthening and sustaining national capacity; 2) prioritizing global over country-specific initiatives undermines locally defined goals and existing programs; 3) inadequate contextualization contributes to misaligned priorities and unsustainable program outcomes; 4) decision-maker bias creates blind spots that inhibit capacity to address gender and health inequities; 5) power asymmetries influence funding and program decisions in-country; 6) donor funding structures pose limitations to program sustainability and impact downstream; 7) program fragmentation inhibits holistic and long-term country planning; 8) decision maker reliance on flawed and incomplete data perpetuates inequities; and 9) overemphasis on donor-prioritized data and evidence creates undue burden on health workers and perpetuates fragmented data systems.

These syndromes reflect the views and perceptions of the co-creation participants ^33^. We recognize that system syndromes vary based on country and/or donor context. Therefore, the syndromes should not be interpreted to reflect the behaviors of specific actors, nor as manifesting in all contexts and initiatives. There are also overlapping concepts between syndromes that lead to different drivers depending on the narrative in focus. The authors encourage readers to consider these syndromes as occurring concurrently and influencing one another within one system.

**Box 1. Reading syndrome maps**

Each syndrome is depicted visually (see Figure 2– Figure 10) and accompanied by a written description of its concept. On the syndrome maps, each circle represents a driver in the system. The lines that connect one circle to another suggest a causal relationship in the direction of the arrow. The thicker arrows highlight feedback loops (i.e., cyclical clusters of drivers that reinforce each other, amplifying their effect and perpetuating a set of system behaviors). Since the initiative focused on the interactions between donors and country recipients, the maps are spatially arranged by drivers that can be observed in three broad contexts: the donor space (left); the country space (right); and the interaction space (middle). The spatial arrangement of these drivers and their connections are designed to facilitate reflection and understanding of how system drivers interact and where within the system they may originate.

*1. Reliance on external implementing partners undermines strengthening and sustaining national capacity*

This syndrome ( Figure 2) depicts several interrelated challenges that pose barriers to retaining sufficient human capacity within a country’s health system. Donors often seek partnerships with established international implementing organizations that are familiar with their structures and have infrastructure (e.g., financial systems, necessary clearances, pool of human resources, databases) to deliver programs according to donor requirements. In such instances, local organizations and expertise may be relegated to minor roles or not used, which limits funding opportunities for local organizations and talent. This in turn perpetuates a dependency on and donor preference for international experts and organizations, which can erode opportunities for local capacity strengthening.

These factors also contribute to a perceived status divide between international and local talent. Local experts may seek employment opportunities with international organizations where they are likely to receive greater remuneration, opportunities, and recognition. Many of these organizations hire local experts who have evolved into international experts, where they are able to further their career development trajectories and gain more experience. This contributes to high turn-over and a drain of local capacity in a country’s health system.

Compounding this, staff assigned to attend donor-sponsored training programs may not be those who would directly benefit from the training or use the skills in their job, limiting the effectiveness and sustainability of such trainings. Selection of staff for training can be influenced by myriad factors including seniority, availability, and/or the incentive of collecting training stipends. For instance, donors -funded programs may not be able to compensate government staff for the additional responsibilities associated with their programs or projects. This, in conjunction with low government remuneration, may contribute to government staff attending donor-funded trainings that they may not need in order to collect stipends.

*2. Prioritizing global over country-specific initiatives undermines locally defined goals and existing programs*

**Figure 3.**
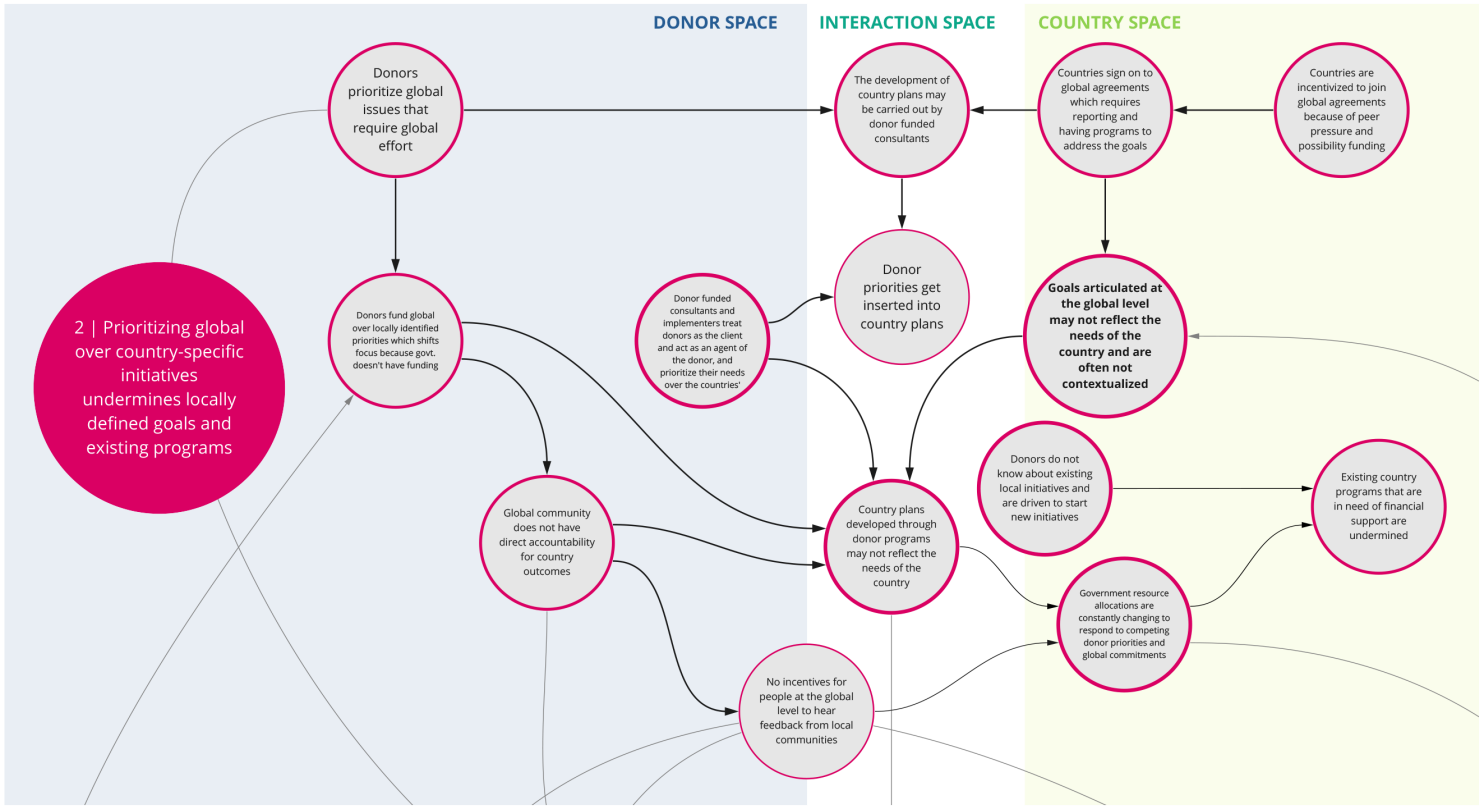
Syndrome 2: Prioritizing global over country-specific initiatives undermines locally defined goals and existing programs.

Co-creation workshop participants identified a tension between global and local priorities ( Figure 3). For instance, donors tend to align with and fund globally defined priorities, which in turn influences country health planning and domestic resource allocation. Countries may join global initiatives or programs because of the associated funding prospects and opportunities. However, goals articulated at the global level may not fully reflect the needs or priorities of individual countries. The corresponding implementation, monitoring, and reporting requirements of these initiatives (e.g., metrics for progress at the country level) can shift a country’s health agenda towards global priorities, which are not often contextualized to the country, and may undermine locally defined goals and existing programs. This also tends to divert scarce resources, including human, to operations focused on targets for global instead of national agendas. This may be further complicated by shifting guidance and concepts at the global level, which countries then must reflect in policy and practice. Overall, this tension between local and global priorities is perpetuated by the lack of incentive or mechanism for the global community to seek feedback from local communities.

*3. Inadequate contextualization contributes to misaligned priorities and unsustainable program outcomes*

**Figure 4.**
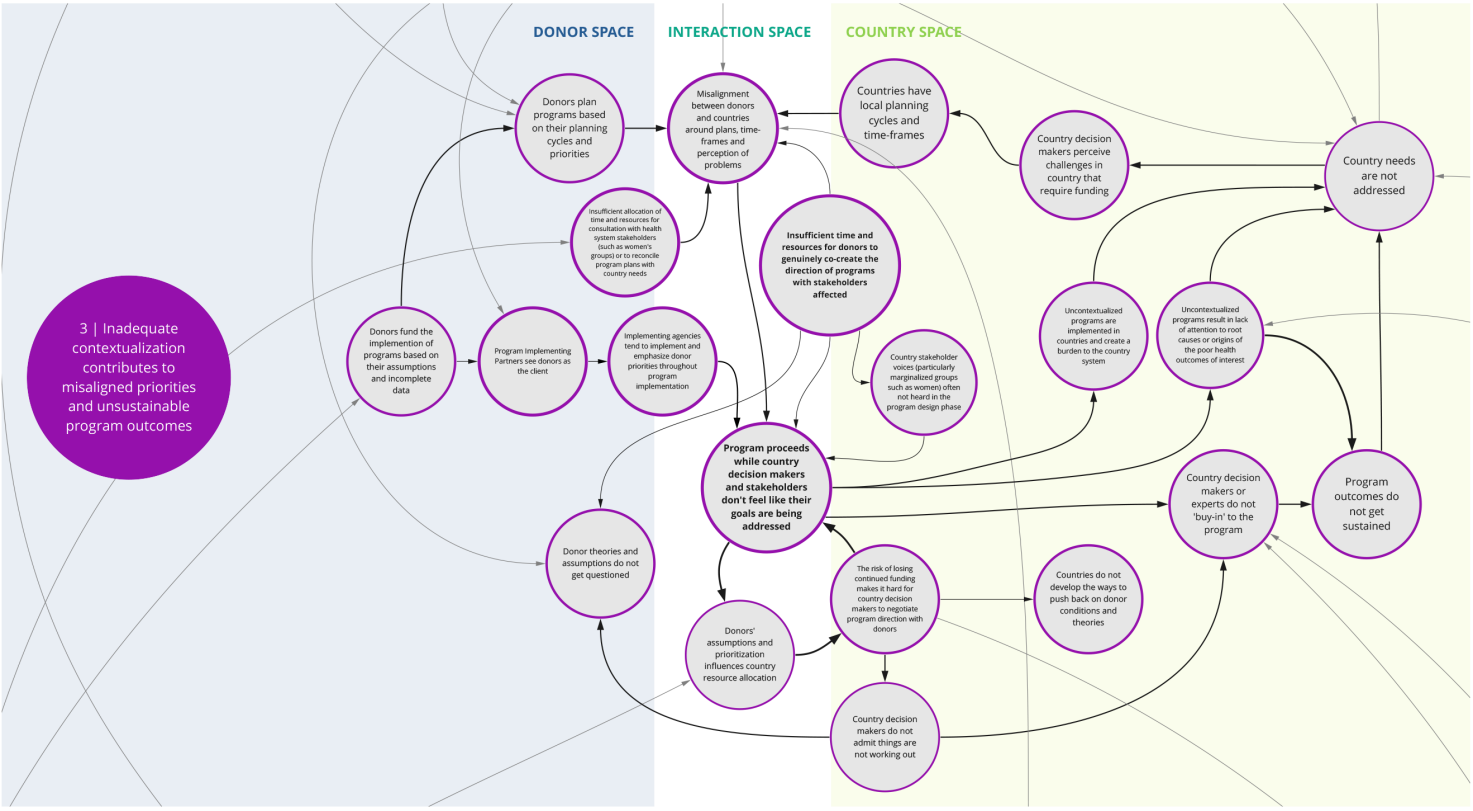
Syndrome 3: Inadequate contextualization contributes to misaligned priorities and unsustainable program outcomes.

This syndrome ( Figure 4) depicts the recurring dynamic where insufficient contextualization of program goals and design contribute to misalignments between donor and local priorities. Co-creation workshop participants largely attributed this to donor-funded programs being based on assumptions about local needs and appropriate approaches, as well as donor’s own planning cycles and priorities. Compounding this is that implementing partners typically act as though the donor is ‘the client,’ which can further undermine country voices and needs.

Participants discussed that a more rigorous and accountable contextualization process could alleviate such issues. However, some participants noted that insufficient allocation of time and resources to fully engage with a wide range of health system stakeholders (including civil society groups and community representatives) inhibits sufficient contextualization of programs. Specifically, there is rarely adequate representation of local experts and marginalized community member perspectives in the design process. Under these circumstances, the priorities of donors and perspectives of a few representatives who are high in the health system dominate program focus.

Compounding this challenge are the ever-shifting dynamics of a given context or health system. The analysis informing contextualization is sometimes just a snapshot in time. Considering that there will be a lag from program conception to implementation, the context may be out of date or inaccurate by the time a program begins. Once a program begins, its ability to change direction or adjust to circumstances may be limited. Participants suggested that a contributing factor to program rigidity is that stakeholders may not have opportunities or the impetus to challenge program decisions, in part due to the fear of losing funding. This may lead to a lack of ‘buy-in’ among country stakeholders and contribute to unsustainable program outcomes.

*4. Decision-maker privilege creates blind spots that inhibit capacity to address gender and health inequities*

**Figure 5.**
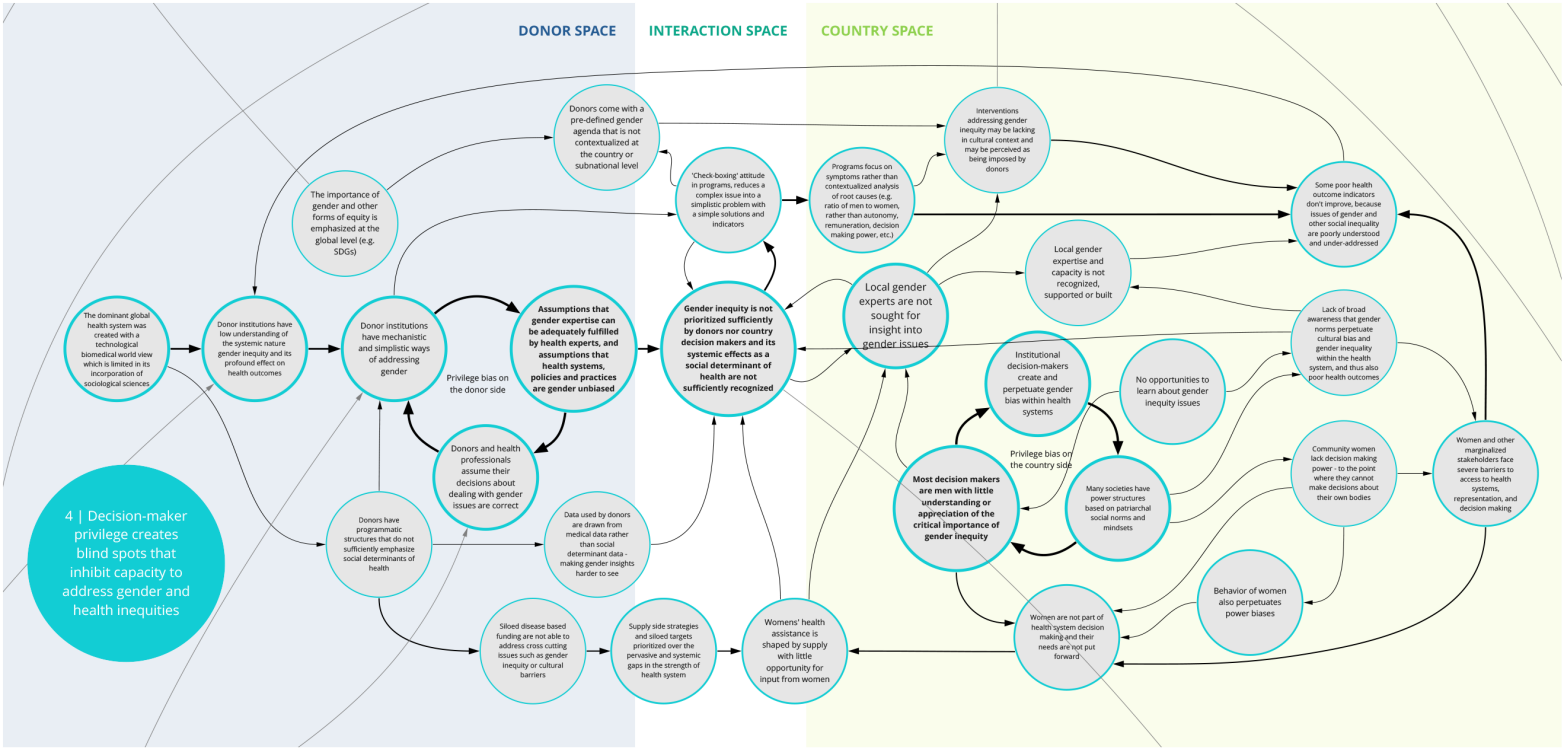
Syndrome 4: Decision-maker privilege creates blind spots that inhibit capacity to address gender and health inequities.

Co-creation workshop participants identified a set of feedback loops contributing to gender bias and other forms of inequities that perpetuate poor health outcomes ( Figure 5). Donor and national health system decision makers’ assumptions about health care are shaped by their own experiences. Social systems of unearned privilege and power influence how health care is planned, funded, and evaluated. Without the opportunity for internal reflection, donors and health care decision-makers may be blind to their own biases and assume their decisions about dealing with gender issues are correct. This can contribute to a limited understanding of the way that health systems are biased and discriminate against many groups. Such preconceptions can lead to programs that reinforce (rather than dismantle) inequities.

For instance, donor institutions often rely on overly generalized or mechanistic approaches to addressing gender inequity. A ‘check-box' approach reduces complex issues of power into simplistic solutions that are not adequate for addressing system-wide disparities. Furthering this, donors and country decision makers tend to overlook and fail to resource the input and expertise of local gender experts in planning and implementing health programs. Insufficient awareness of gender inequities and lack of self-reflection about privilege on the part of decision-makers and implementers play a significant role in gender-blind health systems and health programs. Donors and country decision-makers rarely prioritize system-wide solutions and resources for overcoming gender inequity.

*5. Donor funding creates power imbalances that deter country decision-makers from pushing back on unsuitable funding arrangements*

**Figure 6.**
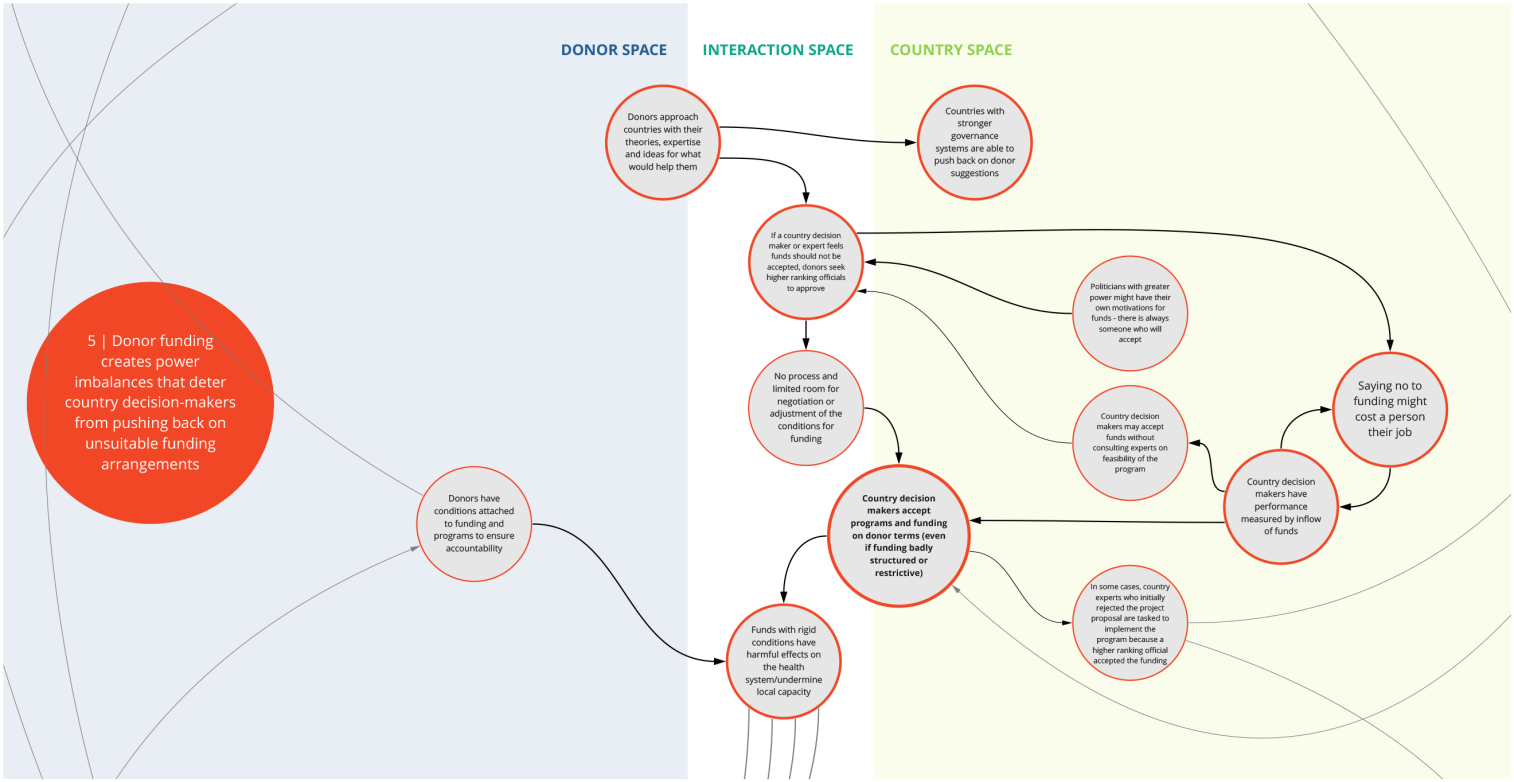
Syndrome 5: Donor funding creates power imbalances that deter country decision-makers from pushing back on unsuitable funding arrangements.

This syndrome ( Figure 6) highlights the power dynamics in program decision making posed by the funder-recipient dichotomy. Specifically, participants discussed how institutions and structures in some countries are set up to prioritize and incentivize acceptance of external funding. This can discourage country decision makers from negotiating terms and conditions when funding opportunities are made. In some instances, this may contribute to inefficient use of funding or may even lead to unintended harm such as inadvertently deprioritizing important health needs that have less funding ties.

Participants also described situations where a donor organization may approach a country agency with program ideas or priorities tied to a set of funding conditions. In some instances, if local subject-matter experts push back on the feasibility of implementation or the need of a given program based on country priorities, the donor organization may ignore this advice and ask decision-makers with greater power in the country to accept the program. Such occurrences can undermine the trust of local agencies and sustainability of the investment.

*6. Donor funding structures pose limitations to program sustainability and impact downstream*

**Figure 7.**
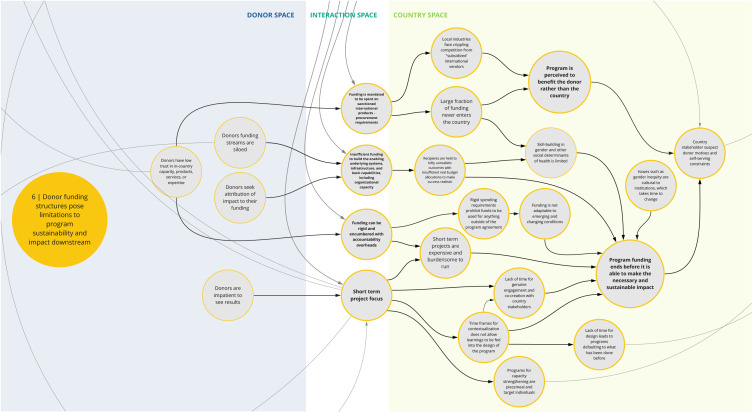
Syndrome 6: Donor funding structures pose limitations to program sustainability and impact downstream.

Co-creation workshop participants described how rigid donor funding structures and conditions can impede program success, limit the funding that is actually spent in the country, and erode trust in donor motivations ( Figure 7). This syndrome highlights several challenges that can manifest from donor funding structures and undermine capacity-strengthening outcomes. For instance, procurement requirements and costs favor international providers and products, which can cripple local industry and limit funding spent in country. Additionally, insufficient funding for long-term, crosscutting, and system-based efforts impede strengthening of institutional capacity and favorable conditions for sustained success. Compounding this, rigid funding restrictions and bureaucracy can prohibit program adaptability and lead to inefficiencies in spending, including high overhead costs. Participants also noted that short-term project cycles contribute to insufficient contextualization and stakeholder engagement and lack of long-term thinking to sustain effective projects. In these cases, funding and programmatic structures may not achieve optimal or intended outcomes, and in some instances have unintended negative consequences. 

*7. Program fragmentation inhibits holistic and long-term country planning*

**Figure 8.**
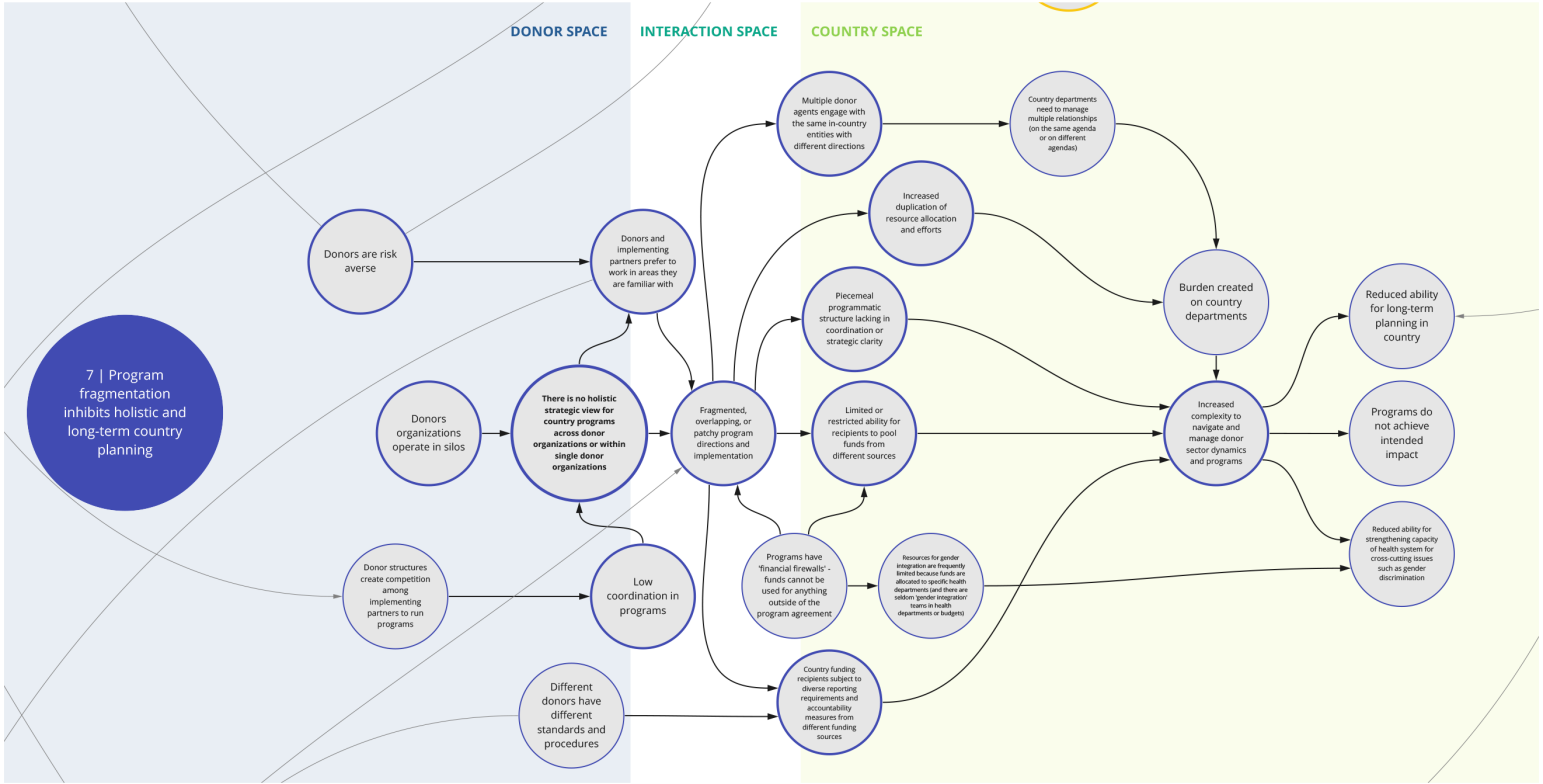
Syndrome 7: Program fragmentation inhibits holistic and long-term country planning.

This syndrome ( Figure 8) highlights the factors contributing to and challenges posed by fragmented health programming and priorities. For instance, donors may create focused funding pools segmented by disease area to be able to scale up assistance to more countries. This may lead to mismatches with government priorities and country health needs. Donors may also be averse to expanding beyond familiar partners, geographies, and/or strategic focus areas due to potential risks in successfully implementing programs. Together these factors inhibit a holistic strategic view and planning for country programs within and across donor organizations, which contributes to overlapping and fragmented implementation. Compounding this, donor structures incentivize competition, which may thwart coordination and knowledge sharing among implementing partners. Additionally, certain funding structures and 'financial firewalls' prohibit use of funds outside specific program requirements.

This fragmentation of programming and the varying requirements and procedures imposed by different funding sources contribute to an increased burden on country recipients to follow diverse data collection, reporting and accountability requirements and navigate varying donor dynamics and programs. Overall, these challenges disrupt programs, hinder the ability for long-term planning, and limit efforts and incentives to strengthen systems and overcome crosscutting challenges.

*8. Decision maker reliance on flawed and incomplete data perpetuates inequities*

**Figure 9.**
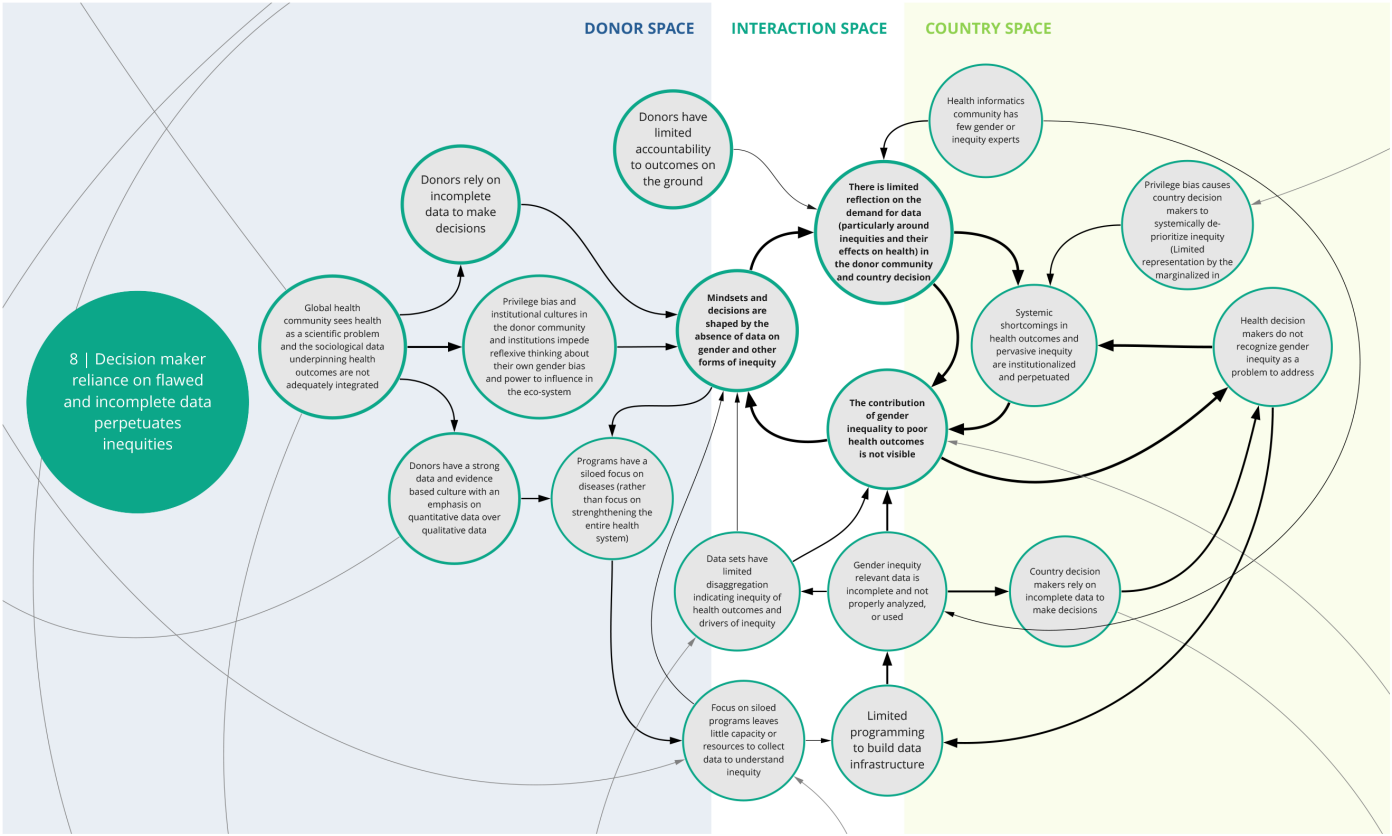
Syndrome 8: Decision maker reliance on flawed and incomplete data perpetuates inequities.

This syndrome ( Figure 9) articulates the problem that donors and government/country-level decision makers face in relying on flawed or incomplete data, which ultimately perpetuates gender discrimination and other inequities. Co-creation workshop participants said that in health programs, data are rarely collected and disaggregated in ways that provide nuance to highlight gender inequities and their contribution to poor health outcomes. There are few opportunities for input by gender experts and populations affected by gender discrimination and bias to inform the collection, analysis, and/or use of routine data for health program decisions. In addition, donors and country-level decision-makers rarely track or use data relevant to social determinants of health (such as son preference, and women’s mobility and ability to make decisions about their bodies) to plan or assess health investments. Thus, health investment decisions are based on incomplete data and may lead to ineffective programs and contribute to systemic inequities being institutionalized and perpetuated.

*9. Overemphasis on donor-prioritized data and evidence creates undue burden on health workers and perpetuates fragmented data systems*

**Figure 10.**
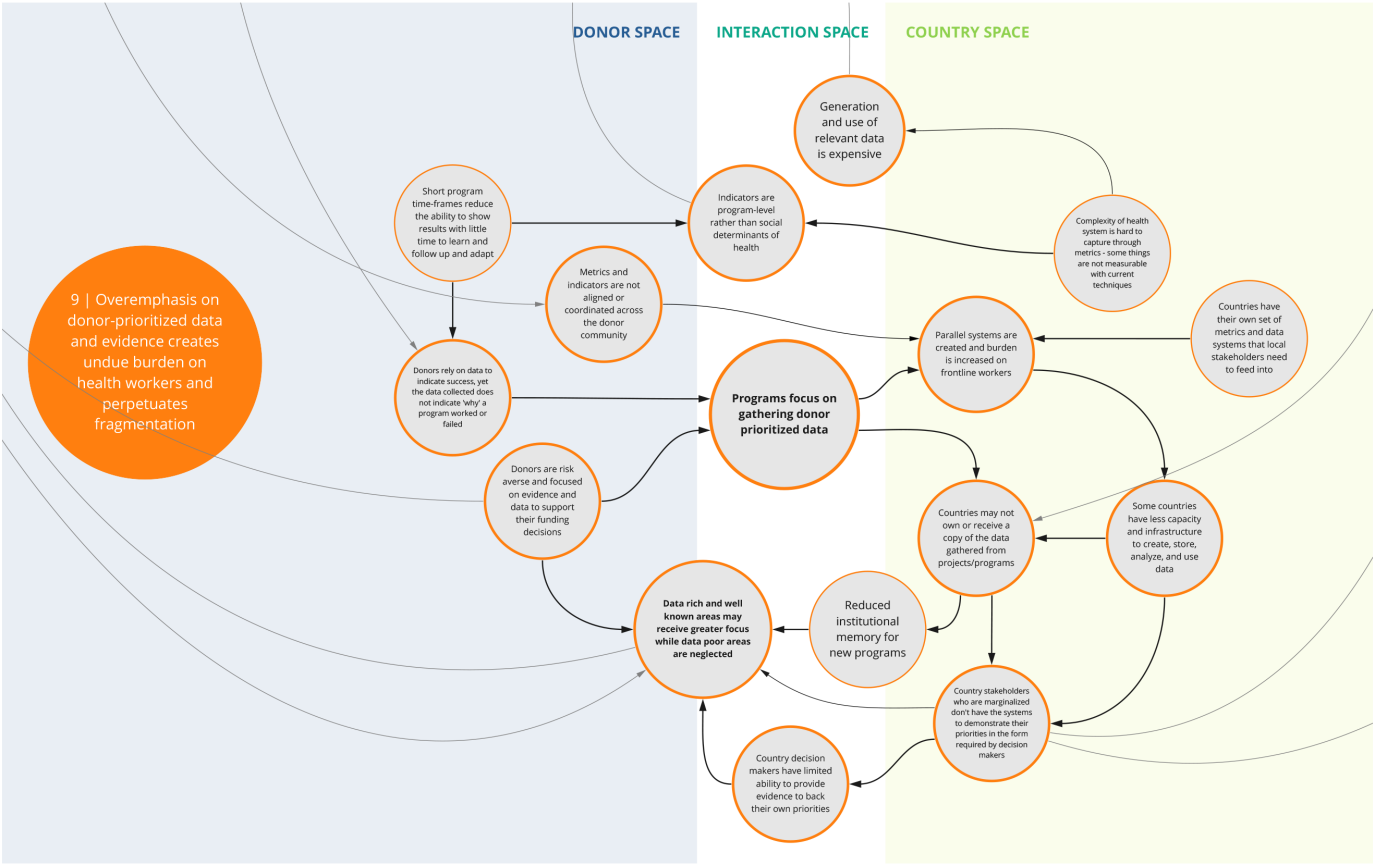
Syndrome 9: Overemphasis on donor-prioritized data and evidence creates undue burden on health workers and perpetuates fragmented data systems.

This syndrome ( Figure 10) depicts key challenges related to decision making and prioritization of data and evidence. For instance, participants described how programs often emphasize donor-prioritized data needs to showcase success and validate their funding decisions, meaning that programs often focus on gathering donor-prioritized data. In some instances, donors and their implementers set up parallel systems to collect data specific to their area of priority instead of using existing country mechanisms and/or investing in more robust health management information systems and data collection tools. This, with the emphasis on program-level indicators and the short-term nature of funding, perpetuates misalignment across programs and often increases the reporting burden on local counterparts. Fragmented data systems may impede a government’s ability to effectively use data. In addition, the emphasis on immediate results contributes to insufficient focus on issues that are harder to measure, undermining the ability to drive longer-term health and/or system outcomes.

### Syndromes in the context of the critical shifts

While this paper mainly focuses on elaborating the specific dynamics within each syndrome, it is important to understand that drivers across the syndromes are inextricably and dynamically interconnected. To transform the system outcomes toward the Critical Shifts for CS, the syndromes must be addressed in tandem, as siloed efforts or interventions are unlikely to disrupt these interconnected systemic challenges.

## Discussion

There is recognition within the global health community that the current development assistance for health model is not producing the intended outcomes in LICs ^27, 34, 35^. Established approaches to problem solving for global health challenges (i.e., isolating and addressing specific problems within a given system), have produced singular, siloed solutions that fail to acknowledge interconnected complexities and fully address underlying causal issues ^30^. Over the last 20 years, many programs and global agreements that outline ways for more effective aid delivery have been established to reconcile these challenges ^14^. Despite these collective efforts, the global health community continues to struggle with reforming development assistance amidst structural complexities and a growing number of actors ^18^.

A characteristic of complex systems is that no actor is intending harm; they are driven by the forces and structures of the system, yet the sum effect can lead to suboptimal outcomes ^36^. Hence, convening a diverse group of global health actors to engage in dialogue and collaboratively develop a holistic understanding of the system is critical to improving the system. This initiative convened donors and country actors to examine the complex development assistance for health system and, using an innovative systems transformation co-creation process, identify barriers impeding the realization of a better, more sustainable aid model as illustrated by the Critical Shifts for CS.

### Enduring challenges in development assistance for health

Through this diagnostic process, we identified nine system syndromes that shed light on structural and causal patterns that manifest between donors and recipient countries. These interconnected syndromes indicate challenges that hinder development assistance goals and that highlight the interdependencies, feedback loops, and vicious cycles that play out throughout the system. While the maps do not reflect how challenges manifest in all cases, the syndromes that emerged from this work are widely reflected in the literature. There are many instances in the literature that point to shortages in health human resources and challenges to deploying and retaining a capacitated health workforce in LMICs ^37– 40^. The effects of misalignment between external funding and country priorities, time-limited programs and rigid funding/implementation structures, and enduring system fragmentation, as illustrated by the vast number of vertical health programs and reporting systems, are also well documented ^4, 18, 27, 41– 43^. Spicer *et al.* ^18^ indicate that these enduring challenges to development assistance reform undermine the effectiveness of health programs and threaten the achievement of better and sustained health outcomes.

### Manifestation of power dynamics across the system syndromes

A driving force across the syndromes is the manifestation of power imbalances between system actors. There is growing recognition in the literature on power and its role in shaping health policy and systems ^44– 47^. In complex adaptive systems like global health, power manifests in multifaceted ways and permeates all levels of the system ^45^. As illustrated in syndromes 1, 4, and 8 ( Figure 2, Figure 5, and Figure 9) and confirmed in the literature, power imbalances between development assistance actors contribute to ongoing health systems challenges, including the perpetuation of inequities and the hampering of local capacity strengthening ^7, 18, 44^. Power dynamics also intrinsically relate to the misalignment of priorities between donor and/or global actors and countries ^18^. This can lead to a lack of buy-in from local actors and communities and perpetuate system fragmentation through siloed health programming, as reflected between syndrome maps 2, 3, 6, and 7 ( Figure 3, Figure 4, Figure 7, and Figure 8). This asymmetry in power between donors and country actors can undermine existing country priorities and programs, and perpetuate inflexible program models that are difficult for countries to adapt for local conditions. This can create downstream dilemmas as countries work to retrofit a programmatic model that may not be contextually appropriate or produce the intended or needed outcomes in the time allocated. Furthermore, as explored in syndrome 5, funding power also influences how decisions are made ( Figure 6). To this point, Olusoji ^48^ highlights that in some cases a fear of “annoying donors and losing funds” dissuades country officials from pushing back on TA efforts, particularly those tied to bilateral programing.

Reformation of this system requires solutions that account for these intertwined complexities. The syndrome maps highlight that change is needed not only in how we structure and implement global health programming and prioritize goals, but in how system actors interact. This will require intentional shifts in the structures, norms, and mindsets that govern global health aid models.

### Promising practices to disrupt the syndromes

Progress toward a more effective aid partnership model is under way. There are promising models of donor accountability to funding recipients and program beneficiaries that work to correct power imbalances ^49^. There is increased demand for models of assistance that emphasize building resilient health systems and prioritize more equitable and locally grounded global health practice ^8, 9, 50^. The State of Commitment to Universal Health Coverage (UHC) Synthesis report (2021) recommends that approaches to achieving UHC should involve investment in resilient and equitable health systems, increase the participation of non-state and non-health sector actors, and use multidimensional approaches to ensure no one is left behind ^51^. Additionally, there are growing calls for investing in local human and organizational capacity through direct grants to local institutions and/or country governments, which will be key to long-term capacity and sustainability of health investments ^52^.

The notion of involving more diverse groups of stakeholders is reinforced across the literature. Ottersen ^53^ points to the need for better connections and more opportunities for dialogue between state and non-state actors to promote transparency and accountability during decision-making processes that affect health policy. These types of multi-stakeholder accountability platforms are needed to ensure that there is more consistent attention to issues beyond the immediate interests of those who hold relative power (e.g., donors/funders, politicians). Innovative co-creation processes like human-centered design and systems thinking can help balance these dynamics and allow for more collaborative design and user-centered programs in global health ^54, 55^. These processes shift the power for funding priority and approach decisions to country partners and create opportunities for dialogue with people most affected by program policies and implementation ^7, 56^. Effective engagement of diverse voices throughout the design and implementation process can support collaboration, contextualization, and alignment on priorities ^54^.

The COVID-19 pandemic has increased calls for reform of broader global health frameworks and governance to support health-system resilience. Lal *et al*. ^9^ point to a need for a “reimagined framework for global health” that centralizes equity and a rights-based approach to health governance; formulates indices that can assess health-system governance resilience and account for explicit and implicit power dynamics; integrates global health security competencies into UHC; and prioritizes unified health financing and innovative domestic funding sources.

While there is no simple way to transform systems, the aforementioned examples are a starting point to advance thinking and innovation on the type of synergistic reforms needed to reframe and restructure the donor-recipient relationship.

### Leveraging the syndromes as a diagnostic tool

The syndromes identified in this initiative (and others that may be identified in the future) can be used to accelerate progress toward achieving the vision of the Critical Shifts for CS by helping stakeholders overcome implicit biases about the efficacy of the system and acknowledge and relinquish their role in perpetuating the syndromes. To this end, the syndromes can expand our understanding of challenges and support the formulation of actions and interventions to change the system. Funding partners could use the syndromes as a starting point for transparent dialogue and diagnostic analysis with country stakeholders to improve efficacy and sustainability of future health investments. For instance, the maps can be used to explore how these syndromes manifest in a specific program or country context and facilitate a common understanding of the broader systemic challenges. The syndromes could also be incorporated into monitoring, evaluation, and learning processes to support identification of issues in program models and implementation. Furthermore, the maps can be used to promote more holistic thinking in investment strategies and consideration of how funding models and approaches may perpetuate or contribute to the syndromes. This can increase awareness of the unintended consequences of power dynamics associated with development assistance.

It is also important to recognize that system transformation is not a singular event; ongoing conversations and iterative learning are essential to prompt reflection and collaboration among all global health partners. While we have highlighted opportunity areas for donors based on our findings, it will be important to continue to engage actors in co-creating a portfolio of actionable and synergistic solutions to achieve the desired system transformation.

### Limitations

The Systems Acupuncture method focuses on co-creating a shared understanding of a system. The findings presented in this paper are drawn from the rich insights, lived experiences, and perspectives of those who participated in the co-creation process. The individual drivers and dynamics should be interpreted as examples of how such challenges have manifested in individual contexts—not a generalizable mapping of the entire system of development assistance for health. Given the substantial variation across donor funding and operational modalities as well as recipient country contexts, this mapping may not reflect all donor-country interactions. Rather, this mapping should be viewed as a high-level perspective of how external funding for health in LICs influences a highly dynamic and complex system. Furthermore, the bias of the team may have influenced syndrome analysis and framing. We sought to mitigate these biases by using the original participant wording where possible and seeking periodic content review from participants throughout the process.

It should also be noted that finding solutions was beyond the scope of this initiative. While we have presented some promising practices based on the literature, further co-creation will be an important next step to explore solutions and advance progress.

## Conclusion

This paper offers the Critical Shifts for CS as a new paradigm for better, more sustainable development assistance for health and presents a system diagnostic that articulates the causal dynamics and barriers to their realization. These syndromes can encourage reflection and dialogue among donor institutions and country stakeholders. The use of such a mechanism to question institutional norms, in a field mired by legacy systems and historic imbalances, is a first step to identifying and reconciling the systemic nature of normative approaches that fail to close existing gaps in health outcomes, despite continued investments.

The holistic nature by which we view systemic barriers and their relationships can help system actors think differently about complex issues and may shed light on solutions that have the ability to disrupt the system. While the specific relationships and drivers will vary across settings, the results garnered under this initiative serve as a starting point to further co-create an understanding of the system in different contexts. We call on actors across global health to leverage these findings and reflect on how global health can be better governed, funded, and implemented to achieve the vision of the Critical Shifts for CS. Amidst the COVID-19 pandemic, with its immense challenges, our global health community has an opportunity to build accountability commitments to ensure solidarity and shared responsibility in transforming development assistance to improve and sustain equitable health outcomes.

## Data Availability

Zenodo: Critical barriers to sustainable capacity strengthening in global health: A systems perspective on development assistance. https://doi.org/10.5281/zenodo.6612438 ^33^ This project contains the following underlying data: Draft Syndrome maps_compiled.pdf Draft Syndrome Maps_individual.pdf Raw Data_ April 2021 Co-Creation Workshop.pdf Data are available under the terms of the Creative Commons Attribution 4.0 International license (CC-BY 4.0).

## Footnotes

^i^ For the purpose of this initiative, we use the term donor to refer to institutions that provide external funding for development assistance in health. While varying terminologies describe those in the funding space, we continue to use donor for clarity and consistency, as this is how we framed the conversation with co-creation participants throughout the process.

^ii^ Ethiopia (2 participants), Ghana (4), India (2), Kenya (5), Malawi (3), Mexico (1), Mozambique (1), Nepal (2), Nigeria (9), Uganda (2), United States (6), Zimbabwe (2), Zambia (2).
