# Supplementary material for: Critical barriers to sustainable capacity strengthening in global health: a systems perspective on development assistance
Source: Gates Open Res. 2023 Jan 18;6:116. Originally published 2022 Aug 24. [Version 2] doi: 10.12688/gatesopenres.13632.2 (PMC9646484; doi:10.12688/gatesopenres.13632.2)
Supplement: Peer review history

# Peer review: Reviewer response for version 1

**DOI:** 10.21956/gatesopenres.14911.r32617

**Published:** 2022-11-09

**Authors:** Shabina Ariff

**Affiliations:** 1 Department of Pediatrics & Child Health, The Aga Khan University, Karachi, Pakistan

A well written paper that describes the impediments to sustainable improvement to MNCH health across LMIC despite significant investment.

 The design and methods are accurate, however I would suggest describing gender equity amongst the stakeholders and a few examples of success stories that have addressed some of the highlighted barriers.

 The various roles cited of actors and their subsequent contribution to the barriers require more elaboration and why many of these initiatives like Universal coverage can be contextualized and how it can be more effectively implemented.

 Within countries there are intra-collaboration and obstacles as well as potential power games that downplay the interventions and impact.

 Overall a very impressive DAH Evaluation paper.

Is the work clearly and accurately presented and does it cite the current literature?

Yes

If applicable, is the statistical analysis and its interpretation appropriate?

Yes

Are all the source data underlying the results available to ensure full reproducibility?

Yes

Is the study design appropriate and is the work technically sound?

Yes

Are the conclusions drawn adequately supported by the results?

Yes

Are sufficient details of methods and analysis provided to allow replication by others?

Yes

Reviewer Expertise:

I am a public health specialist working around maternal ,newborn and child health.

I confirm that I have read this submission and believe that I have an appropriate level of expertise to confirm that it is of an acceptable scientific standard.

# Response

**Published:** 2023-01-11

**Authors:** Barbara Knittel

**Affiliations:** JSI Research & Training Institute, Inc., USA

Dear Shabina Ariff, 

 Thank you for your thoughtful review and comments. In response to your suggestions to describe gender equity amongst the country participants and offer examples of success stories that address some of the highlighted barriers, we offer the following explanations: 

1. We did not ask co-creation participants to identify their gender as part of the process and therefore have not included this information in the manuscript. Unrelated to your particular comment, but it be of interest to you, we have developed a corresponding manuscript that explores system drivers of gender inequity.
2. The scope of this work was to examine the problem space and barriers to achieving the critical shifts for DAH. This manuscript focuses on articulating those identified barriers and invites the global health community to think through these issues and offer potential solutions. In the conclusion we have offered some broad solutions found in the literature, however, this process envisions co-creating solutions rather than dictating them and, unfortunately, the scope of this work did not extend to solutioning other potential fixes to the barriers identified. 

We would like to thank you for your additional comment,  "The various roles cited of actors and their subsequent contribution to the barriers require more elaboration and why many of these initiatives like Universal coverage can be contextualized and how it can be more effectively implemented." It is well noted and we agree that this is an important step for further exploration. 

 Thank you, 

 Barbara Knittel

# Peer review: Reviewer response for version 1

**DOI:** 10.21956/gatesopenres.14911.r32619

**Published:** 2022-10-10

**Authors:** Neil Spicer

**Affiliations:** 1 Department of Global Health and Development, Faculty of Public Health and Policy, London School of Hygiene and Tropical Medicine, London, UK

**Introduction**

 It would be helpful to clarify the scope of the types of donors being discussed. The line ‘bilateral donors, centrally funded mechanisms, and philanthropic foundations for implementing health program’ wasn’t that clear. Could this be unpacked a bit? – e.g. I wasn’t clear on what are centrally funded mechanisms. Does donors mean the ‘traditional’ bilateral donors if so, which ones are included? Does the analysis include ‘new’ donors such as China? I wasn’t clear if major initiatives such as the Global Fund and Pepfar were being included. And funding via UN agencies. And which foundations.

 Could the authors also briefly reflect on who this paper might be useful to – researchers, donors, LIC country governments?

**Methods**

 Important to add a bit more information on the 41 participants. What were the selection criteria? What countries and (sorts of) organisations do they represent? And a breakdown of types of participants e.g. representing governments of LICs, different types of donors, researchers etc?

**Results**

 The results section presents interesting material. However, it does tend to simplify and generalise issues that vary very substantially between different LICs and between different funders and types of funders e.g. different bilaterals behave in very different ways of working, as do other major funders such as the Gates Foundation, Global Fund, Pepfar and others. This is briefly acknowledged in the Limitations right at the end, but I felt some sense of the substantial variations among countries and donors should be reflected in the Results.

 Many of the ‘syndromes’ described – while clearly identified and described (which is useful) - are well known and widely documented already. And so, it would be nice for this paper to move beyond this – the intro talked about health systems being complex adaptive systems – can this be brought into the analysis? Perhaps also drawing more on the PowerPoint mind maps in the main text – including drawing out some of the most important feedback loops and interactions, and distinguishing between donor space, interaction space and country space?

 Additionally, the paper documents many of the problems with DAH. But it would be nice to see more about the potential solutions to each of the syndromes – and who should be responsible for making changes stemming from the viewpoints of the participants (e.g. should donors be the ones changing – and/or what can LICs do about this issues?)

Is the work clearly and accurately presented and does it cite the current literature?

Yes

If applicable, is the statistical analysis and its interpretation appropriate?

Not applicable

Are all the source data underlying the results available to ensure full reproducibility?

Partly

Is the study design appropriate and is the work technically sound?

Yes

Are the conclusions drawn adequately supported by the results?

Yes

Are sufficient details of methods and analysis provided to allow replication by others?

Partly

Reviewer Expertise:

Global health policy and systems research

I confirm that I have read this submission and believe that I have an appropriate level of expertise to confirm that it is of an acceptable scientific standard, however I have significant reservations, as outlined above.

# Response

**Published:** 2023-01-11

**Authors:** Barbara Knittel

**Affiliations:** JSI Research & Training Institute, Inc., USA

Dear Neil, 

 Thank you for your thoughtful review and comments. Please see below our responses to your comments and/or suggestions.

 "It would be helpful to clarify the scope of the types of donors being discussed. The line ‘bilateral donors, centrally funded mechanisms, and philanthropic foundations for implementing health program’ wasn’t that clear. Could this be unpacked a bit? – e.g. I wasn’t clear on what are centrally funded mechanisms. Does donors mean the ‘traditional’ bilateral donors if so, which ones are included? Does the analysis include ‘new’ donors such as China? I wasn’t clear if major initiatives such as the Global Fund and Pepfar were being included. And funding via UN agencies. And which foundations." 

**Response:**

- In the introduction, we are citing literature focused on the broad landscape of aid e.g. “Low-income countries (LICs) have traditionally relied on funding and technical support from bilateral donors, centrally funded mechanisms, and philanthropic foundations for implementing health programs.” What we mean by “centrally funded mechanisms" is ‘pooled’ or ‘basket’ funds from multiple donors that are commingled in a segregated account by a host government and used to finance specific goods and services. This can also apply to agencies and organizations like WHO, UN, Global Fund, etc.
- Our analysis focused on the broad Development Assistance for Health landscape and did not interrogate specific donors/funders. However, we did have representatives from USAID, World Bank, BMGF, and GFF as part of the co-creation process.

"Could the authors also briefly reflect on who this paper might be useful to – researchers, donors, LIC country governments?"

**Response:**

- Given the systems perspective, this paper is intended for the broad global health community and all actors involved in DAH systems, which is inclusive of donors/funders, implementing partners, governments, researchers, etc. 
  - We say in the introduction “This paper seeks to inform the ongoing discussion on development assistance reform and support the identification of key actions at project, country, regional, and global levels that facilitate investments to sustain local development and build resilient, equitable health systems.” 
  - We also note in the conclusion “These syndromes can encourage reflection and dialogue among donor institutions and country stakeholders.”

"Important to add a bit more information on the 41 participants. What were the selection criteria?"

**Response:**

- We have updated the methods to provide more clarity and details for participant selection. The updated version of the manuscript will include the following under the Methods section: "The secretariat and IAWG members used purposive sampling to identify actors within their networks who could bring diverse perspectives on how health funding and TA is structured at various levels and how donor processes, models, and norms constrain or amplify health system capacity strengthening and sustainable health outcomes. Participants were selected to ensure diversity in background, institutional affiliation, geography and perspectives in order to co-create a systems view."

"What countries and (sorts of) organisations do [the participants] represent? And a breakdown of types of participants e.g. representing governments of LICs, different types of donors, researchers etc.?"

**Response: **

- The countries represented by the participants are included in a footnote (footnote, ii) and their broad organizational representation (i.e. country-based government, civil society, implementers, private sector, and academia) is included in the text. We have not included the names of specific organizations as we did not seek participant approval to include their organizational names.
  - *Footnote ii: Ethiopia (2 participants), Ghana (4), India (2), Kenya (5), Malawi (3), Mexico (1), Mozambique (1), Nepal (2), Nigeria (9), Uganda (2), United States (6), Zimbabwe (2), Zambia (2).*

"The results section presents interesting material. However, it does tend to simplify and generalise issues that vary very substantially between different LICs and between different funders and types of funders e.g. different bilaterals behave in very different ways of working, as do other major funders such as the Gates Foundation, Global Fund, Pepfar and others. This is briefly acknowledged in the Limitations right at the end, but I felt some sense of the substantial variations among countries and donors should be reflected in the Results."

**Response: **

- This is acknowledged at the beginning of the results section: “These syndromes reflect the views and perceptions of the co-creation participants. We recognize that system syndromes vary based on country and/or donor context. Therefore, the syndromes should not be interpreted to reflect the behaviors of specific actors, nor as manifesting in all contexts and initiatives.” 
- This mapping seeks to provide an overarching view of the global health aid system to highlight broad systemic barriers. Unfortunately, it was not within our scope to explore specific donors or countries. However, in the conclusion we suggest this could be done as a next step in specific contexts. While our analysis was limited to the views/ experiences/ perspectives of the included co-creation participants, we are confident these results represent challenges that many country actors face and the way many donors behave. 

"Many of the ‘syndromes’ described – while clearly identified and described (which is useful) - are well known and widely documented already. And so, it would be nice for this paper to move beyond this – the intro talked about health systems being complex adaptive systems – can this be brought into the analysis? Perhaps also drawing more on the PowerPoint mind maps in the main text – including drawing out some of the most important feedback loops and interactions, and distinguishing between donor space, interaction space and country space?"

**Response: **

- While we agree that the syndromes overall present known challenges, the value of this process was co-creating an understanding of how these challenges manifest in the system (as presented through the visual maps in the results section)
- Given the complexity and interrelated factors influencing these challenges, the purpose of this exercise was to create a visual depiction of the system through visual mappings to illustrate connections, feedback loops, and vicious cycles. These visual maps represent the core of our results and we encourage readers to spend time reviewing the maps, alongside the text descriptions as it is challenging to capture all the details in written prose.
- We acknowledge that an important future step is to contextualize these findings in specific settings and further analyze key drivers and feedback loops in those contexts to inform action. While we were not able to do that as part of this exercise, we note that our findings can be leveraged as a starting point for further analysis or contextualization. 

"Additionally, the paper documents many of the problems with DAH. But it would be nice to see more about the potential solutions to each of the syndromes – and who should be responsible for making changes stemming from the viewpoints of the participants (e.g. should donors be the ones changing – and/or what can LICs do about this issues?)"

**Response: **

- The scope of this work was to examine the problem space and barriers to achieving the critical shifts for DAH. The manuscript focuses on articulating those identified barriers and invites the global health community to think through these issues and offer potential solutions. In the conclusion we have offered some broad solutions found in the literature, however, this process envisions co-creating solutions rather than dictating solutions and, unfortunately, this scope of work did not extend to solutioning other potential fixes to the barriers identified. The hope is that this analysis of the problem space will facilitate dialogue and eventually co-created solutions.

Respectfully, 

 Barbara Knittel

# Peer review: Reviewer response for version 1

**DOI:** 10.21956/gatesopenres.14911.r32429

**Published:** 2022-09-26

**Authors:** Peter Eerens MD MPH

**Affiliations:** 1 Living Health Systems, Brussels, Belgium

The authors report on an in-depth exercise to sample an extensive set of drivers that a representative selection of stakeholders of 13 participating countries considered relevant in the system analysis of the dynamics that hinder the achievement of the desired outcomes of critical shifts for capacity strengthening. “Critical shifts for capacity strengthening”, capitalized by the authors as a branded concept, is a vision crafted in an earlier initiative and adapted by the research team to a focus on capacity strengthening, while highlighting also important power and gender dimensions. This vision is also used as a guiding framework when applying the system diagnosis. The research team bundles the very rich set of findings in nine system syndromes. Their display and description constitute the body of the report.

 The reader of this report should be warned. This journal article is probably no match for the creative bursts that occurred during the actual conversations, discussions, sketches, visualisations, drawings etc. that took place during this 12-month co-creation process.

 The number of drivers identified are a valid reflection of the multi-dimensional nature of the overall challenge that improvement of inter-country collaboration for health poses. The reported findings are not really a surprise. As the authors mention, the existing literature points to most of the identified drivers, very often to their relationships, and equally often to how they impact on country priorities and programs.

 Is there thus a merit to this exercise? Absolutely, there is. Although complex systems-based approaches in exploring large scale social systems such as health systems are becoming mainstream, there is no unique analysis that will capture all their complexities. The reader might be helped in appreciating this by recognizing the challenge that multi-dimensionality poses to the study of such systems.

 In my view, the authors struggle somewhat with this. Dimensionality reduction is essential to our ability to grasp complex systems. That is why we invent frameworks. They reduce the hundreds or thousands of degrees of freedom of a system to a few dimensions within which we create further hierarchies or clusters. The critical shifts framework contains ten dimensions, each of them being a “shift from …to…”, and each of them containing a granular description of the desirable state. Because of the design of the system analysis, this dimension-rich guiding framework greatly influences the challenges that participants will identify. It is therefore no surprise that once the authors bundle the system drivers in aggregate form under the system syndrome headings, those system syndromes easily map into the original critical shifts, an exercise that the reader can do once the article has been read and studied.

 Thanks to the efforts of the team in bundling different drivers in system syndromes while also dividing them over three spaces ( i.e. donor space, interaction space and country space), we see how syndromes differ in terms of how densely identified drivers occupy these three spaces. All this is food for thought, as the authors suggest in their discussion and conclusions.

 In summary, having read the article several times because of my interest in the subject and in complex systems, I think that the reader needs to understand fully the method as described in the article to understand its strength but also its limitations. In all fairness, the authors insist that there is more to challenges in capacity strengthening than the output of their work suggests, but that what counts is that this material is used in enriching the search of pertinent answers on how to improve what currently exists in the very concrete donor-country situations.

 One suggestion: add a reference to the Systems Acupuncture method.

Is the work clearly and accurately presented and does it cite the current literature?

Yes

If applicable, is the statistical analysis and its interpretation appropriate?

Not applicable

Are all the source data underlying the results available to ensure full reproducibility?

Yes

Is the study design appropriate and is the work technically sound?

Yes

Are the conclusions drawn adequately supported by the results?

Yes

Are sufficient details of methods and analysis provided to allow replication by others?

Yes

Reviewer Expertise:

Public Health; Health Systems

I confirm that I have read this submission and believe that I have an appropriate level of expertise to confirm that it is of an acceptable scientific standard.

# Response

**Published:** 2023-01-11

**Authors:** Barbara Knittel

**Affiliations:** JSI Research & Training Institute, Inc., USA

Dear Peter, 

 Thank you for your thoughtful review and comments. Thank you for raising the issue of dimensionality. The lens we took with the system mapping was to explore the system through the vision of the critical shifts (co-created with country stakeholders) and specifically mapping barriers to achieving these shifts in the system, with an emphasis on donors/funders. We recognize that there are other contextual issues or lenses that the development assistance for health system could be explored through and we encourage others to use our mapping as a starting point. 

 You also suggested that we add a reference to the Systems Acupuncture method. I want to note that this reference is included in the manuscript (ref. #32): Banerjee, B. (2021). The ABC of planetary insecurity: A crisis in need of system acupuncture. Environmental Conservation, 48(2), 71-74. 

 Respectfully, 

 Barbara Knittel
